# Ryanodine Receptor Mediated Calcium Release Contributes to Ferroptosis Induced in Primary Hippocampal Neurons by GPX4 Inhibition

**DOI:** 10.3390/antiox12030705

**Published:** 2023-03-13

**Authors:** Silvia Gleitze, Omar A. Ramírez, Ignacio Vega-Vásquez, Jing Yan, Pedro Lobos, Hilmar Bading, Marco T. Núñez, Andrea Paula-Lima, Cecilia Hidalgo

**Affiliations:** 1 Biomedical Neuroscience Institute, Faculty of Medicine, Universidad de Chile, Santiago 8380000, Chile; 2 Department of Neurobiology, Interdisciplinary Center for Neurosciences (IZN), Heidelberg University, 69120 Heidelberg, Germany; 3 Department of Biology, Faculty of Sciences, Universidad de Chile, Santiago 7810000, Chile; 4 Institute for Research in Dental Sciences, Faculty of Dentistry, Universidad de Chile, Santiago 8380000, Chile; 5 Department of Neurosciences, Faculty of Medicine, Universidad de Chile, Santiago 8380000, Chile; 6 Physiology and Biophysics Program, Institute of Biomedical Sciences and Center for Exercise, Metabolism and Cancer Studies, Faculty of Medicine, Universidad de Chile, Santiago 8380000, Chile

**Keywords:** cell death, ferroptosis, iron, reactive oxygen species, endoplasmic reticulum, lipid peroxidation, oxidative stress, glutathione peroxidase, RSL3, iron chelators, dendritic morphology, calcium signaling, neurodegeneration

## Abstract

Ferroptosis, a newly described form of regulated cell death, is characterized by the iron-dependent accumulation of lipid peroxides, glutathione depletion, mitochondrial alterations, and enhanced lipoxygenase activity. Inhibition of glutathione peroxidase 4 (GPX4), a key intracellular antioxidant regulator, promotes ferroptosis in different cell types. Scant information is available on GPX4-induced ferroptosis in hippocampal neurons. Moreover, the role of calcium (Ca^2+^) signaling in ferroptosis remains elusive. Here, we report that RSL3, a selective inhibitor of GPX4, caused dendritic damage, lipid peroxidation, and induced cell death in rat primary hippocampal neurons. Previous incubation with the ferroptosis inhibitors deferoxamine or ferrostatin-1 reduced these effects. Likewise, preincubation with micromolar concentrations of ryanodine, which prevent Ca^2+^ release mediated by Ryanodine Receptor (RyR) channels, partially protected against RSL3-induced cell death. Incubation with RSL3 for 24 h suppressed the cytoplasmic Ca^2+^ concentration increase induced by the RyR agonist caffeine or by the SERCA inhibitor thapsigargin and reduced hippocampal RyR2 protein content. The present results add to the current understanding of ferroptosis-induced neuronal cell death in the hippocampus and provide new information both on the role of RyR-mediated Ca^2+^ signals on this process and on the effects of GPX4 inhibition on endoplasmic reticulum calcium content.

## 1. Introduction

Cell death is a fundamental part of the regulation of normal tissues and is a key process in different pathological phenomena. Particularly, cell death of specific subsets of neurons plays a decisive role in many chronic neurodegenerative diseases. Due to the importance of neuronal death in neurologic disorders, it is not surprising that a search for “cell death” and “neurons” in PubMed produces over 65,000 results by December 2022. However, despite years of investigation and the identification of pathogenic factors, the mechanisms underlying neuronal cell death are still poorly defined.

Increasing evidence indicates that neurons are susceptible to ferroptosis—a regulated cell death form described in 2012. Ferroptosis, which is morphologically and biochemically different from other cell death modalities, is characterized by the iron-dependent accumulation of lipid peroxides, depletion of the endogenous antioxidant agent glutathione (GSH), oxidative stress, morphological changes of mitochondria, and enhanced lipoxygenase (LOX) activity [1]. The antioxidant enzyme GPX4 has a key role in preventing ferroptosis due to its unique ability to catalyze the reduction of lipid peroxides via a GSH-dependent reaction [2,3,4]. Mechanistically, ferroptosis can be induced by erastin, glutamate, or sorafenib through the inhibition of the amino acid antiporter system X_c_^−^ and the subsequent decrease in intracellular GSH concentrations, or by the direct inhibition of GPX4 by RSL3 [5].

Reports published in recent years imply a possible role of ferroptosis in hippocampal-dependent neurodegeneration due to large amounts of iron detected in the human hippocampus in Alzheimer’s disease (AD) [6]. Concordantly, studies performed in knock-out mice for GPX4 showed iron deregulation, elevated lipid peroxidation, and inflammation, together with behavioral dysfunction and hippocampal neurodegeneration [7]. Moreover, the protein content of ferroportin—the only known transmembrane protein that transports iron out of the cell—is reduced with age in an AD model and in AD patients, and ferroportin knockout causes hippocampal atrophy and memory impairments in mice [8]; these results imply an important role for iron dysregulation and ferroptosis in hippocampal-related diseases. Although in recent years the number of reports on ferroptosis has increased exponentially, not all the mechanisms underlying ferroptosis in neurons have been elucidated.

Recent evidence hints toward a significant role of dysregulated calcium (Ca^2+^) signaling on iron-dependent cell death [9]. Ca^2+^ is a universal second messenger with crucial roles in neuronal functions such as neuronal excitability, activity-mediated gene transcription, synaptic plasticity, and learning and memory; however, when dysregulated, Ca^2+^ signals can promote cell death [10,11,12,13,14]. Accumulating evidence indicates that increased reactive oxygen species (ROS) levels, including those generated by iron, can modulate the function of key components involved in Ca^2+^ signaling [15,16,17,18,19]. For instance, plasma membrane resident Ca^2+^ channels, such as voltage-gated calcium channels and N-methyl-D-aspartate (NMDA) receptors, as well as the two main channels that reside in the endoplasmic reticulum (ER), the inositol 1,4,5-receptor (IP_3_R) and the ryanodine receptor (RyR), are redox-sensitive [15,20,21,22,23,24]. Interestingly, we have previously reported that iron elicits RyR-mediated Ca^2+^ release and mitochondrial fission in primary hippocampal neurons, which probably promote neuronal dysfunctions [20,25].

In recent years, increasing evidence has shown that Ca^2+^ signals participate in neuronal ferroptosis [9]. Recently, the role of ER-mediated Ca^2+^ signals in ferroptosis has been detailed in different cell lines, indicating that luminal Ca^2+^ stores alter the sensitivity to ferroptosis by causing lipid remodeling [26]. However, in neuronal cells the role of Ca^2+^ release from the ER on this process remains unreported. Considering that the ER is the most prominent intracellular Ca^2+^ reservoir and that RyR channels are highly redox sensitive [27], the ROS increase associated with ferroptosis is likely to promote substantial RyR activation, which could cause abnormally high and noxious increases in cytoplasmic and mitochondrial [Ca^2+^]. A recent study in microglia reported that RyR-mediated Ca^2+^ release contributes to ferroptosis induced by nitrogen-doped graphene quantum dots triggering the ER stress response [28]. These findings provide novel evidence on the possible role of Ca^2+^ release from the ER in the execution steps of ferroptosis.

In the present study, we aimed to first establish an in vitro model of ferroptosis in rat primary hippocampal neurons to then evaluate the role of RyR-mediated Ca^2+^ release from the ER on this process. We found that GPX4 inhibition with RSL3 induced dendritic damage, lipid peroxidation, and cell death; preincubation with the ferroptosis inhibitors deferoxamine or ferrostatin-1 reduced these effects. Suppression of RyR-mediated Ca^2+^ release partially protected against RSL3-induced cell death, whereas sustained GPX4 inhibition prevented Ca^2+^ release induced by the RyR agonist caffeine or by the SERCA inhibitor thapsigargin, and reduced RyR2 hippocampal protein content.

## 2. Materials and Methods

### 2.1. Reagents

RSL3 (C23H21CIN2O5) was purchased from MedChem Express (Monmouth Junction, NJ, USA). Deferoxamine mesylate salt (D9533), Ferrostatin-1 (347174-05-4), DMSO (67-68-5), Poly-D-Lysine Hydrobromide (27964-99-4), 3-(4,5-dimethylthiazol-2-yl)-2,5-diphenyltetrazole (MTT) (M5655), Fluo4-AM (F14201), Caffeine (222402), and Triton^TM^ X-100 solution (93443) were purchased from Sigma Aldrich (St. Luis, MO, USA). Ryanodine (1329) and Thapsigargin (1138) were from Tocris (Bristol, UK). Serum-free Neurobasal Medium, B27 Supplement, GlutaMAX^TM^, DMEM powder (12100046), Antibiotics (Penicillin, Streptomycin, 15070063), HBBS (14025092), and DAPI were from Gibco^TM^/Thermofisher Scientific (Waltham, MA, USA). DAKO mounting medium was from Agilent Technologies (Santa Clara, CA, USA). Lipofectamine 3000 and LIVE/DEAD Viability/Cytotoxicity Kit (L3224) was from Invitrogen^TM^/Thermofisher Scientific (Waltham, MA, USA). Bodipy C11 (27086) was purchased from Cayman Chemical (Ann Arbor, MI, USA). Fetal bovine serum (04-001-1A) was from Biological Industries/Sartorius (Goettingen, Germany). Donkey serum (SH30071.03IH25-40) was from HyClone, Cytiva (Logan, UT, USA). Fluorescent Mounting solution was purchased from DAKO/Agilent Technologies (Santa Clara, CA, USA). Trans-Blot Turbo^TM^ 5x Transfer Buffer and Trans-Blot Turbo^TM^ Mini size VDF membranes were from BioRad (Hercules, CA, USA). The shScr-RFP (TR30015) plasmid was purchased from Origene (Rockville, MD, USA).

### 2.2. Antibodies

Specific primary antibodies against MAP2 (MAB3418) and RyR3 (AB9082) were from Merck (Darmstadt, Germany) and against RyR2 (MA3-916/C3-33) from Thermo Fisher/Molecular Probes (Waltham, MA, USA). All secondary antibodies were from Invitrogen^TM^/Thermofisher Scientific (Waltham, MA, USA).

### 2.3. Primary Hippocampal Cultures

Eighteen-day-old embryos from Sprague-Dawley rats were used to prepare primary hippocampal cultures enriched in neurons, as previously described [29,30]. The hippocampal cells were seeded on poly-D-lysine-coated plates. Cultures were maintained in a serum-free Neurobasal medium, containing B27 supplement, GlutaMAX^TM^, and antibiotics for 14–16 days in vitro (DIV) in a humidified 5% CO_2_ atmosphere at 37 °C. In select experiments performed at Heidelberg University, Germany, primary hippocampal rat cultures from P0 pups were prepared as described [31].

### 2.4. Treatments

Primary hippocampal neurons were cultured at 37 °C on poly-D-lysine-coated multi-well plates; 12–15 days later, cells were treated with the ferroptosis inducer RSL3 dissolved in DMSO. Additionally, cultures were treated with the iron chelator deferoxamine (DFO), Ferrostatin-1 (Fer1), or Ryanodine (Rya) at the concentrations detailed in the figure legends. Incubations were performed in medium. DMSO was used as a negative control.

### 2.5. Transfection of Primary Hippocampal Cultures

Primary hippocampal cultures were transfected at 7–8 DIV using Lipofectamine 3000^®^ (Invitrogen) (DNA:Lipofectamine ratio 1:2) with a shScr vector coupled to a red fluorescence protein (RFP) sequence.

### 2.6. Recombinant Adeno-Associated Viral Vector (rAAV)

Viral particles were produced and purified as described previously [32,33]. Primary cultures were transduced at 7–8 DIV with rAAV-GCaMP3-NES or rAAV-mCherry-NLS at a concentration of 1 × 10^8^ viral particles/mL.

### 2.7. Cell Viability

#### 2.7.1. MTT Assays

To analyze cell viability, cultures were incubated in a supplemented Neurobasal medium with MTT, at a concentration of 0.5 mg/mL for 20–30 min to allow MTT reduction to formazan blue by metabolically active cells. The assay was stopped by adding an equivalent volume of 10% SDS solution in 10 mM HCl, followed by incubation overnight at room temperature (RT). The following day, the absorbance at 570 nm was recorded.

#### 2.7.2. Viability/Cytotoxicity Assay Kit

Cell viability was evaluated using the LIVE/DEAD Viability/Cytotoxicity Kit. Briefly, primary hippocampal cultures were gently washed with warm (37 °C) phosphate buffered saline (PBS) and were subsequently incubated with 2 µM calcein-AM ester and 2 µM ethidium homodimer in PBS at RT for 20 min. After washing three times with warm PBS, cells were counted in three random fields in an epifluorescence microscope (Nikon).

#### 2.7.3. IncuCyte Live Imaging Measurement of Cell Viability

Primary cultures were infected on 7–8 DIV with rAAV-mCherry-NLS to induce the nuclear expression of mCherry. After the respective treatments, cells were placed into an incubator with an incorporated IncuCyte S3 live-cell imaging apparatus (Sartorius). Four images per well were acquired in the red channel every hour for 24 h using a 20× objective. Viable cells presented an intense mCherry fluorescence within the nucleus, whereas dying cells showed a decrease in mCherry-fluorescence over time. For the analysis, the IncuCyte Basic Analysis software was used. The background was subtracted, and objects were selected using defined parameters of threshold and area.

### 2.8. Immunocytochemistry

Hippocampal cultures were fixed in 4% formaldehyde for 20 min at RT. Next, cells were gently washed five times with PBS solution and incubated in PBS solution plus 3% donkey serum plus 0.25% Triton X-100 to permeabilize and block the non-specific binding of the antibodies. In the following step, the cells were incubated overnight with the primary antibody MAP2A (1:500), which was dissolved in the same blocking solution. Cultures were then incubated with secondary antibodies conjugated to a fluorescent dye and mounted on slides in a Fluorescent Mounting solution. The mounted slides were stored at 4 °C for later observation. A confocal microscope (LSM 510, Zeiss, Oberkochen, Germany) with a 40-oil immersion objective and excitation by 543 nm laser was used for the MAP2A images. The ImageJ software program (National Institutes of Health, Baltimore, MD) was used to generate z-projections from seven to 12 stacks (1 µm thickness each). The cells of interest were selected by delineation based on MAP2A fluorescence using the ImageJ software program to determine the neuronal soma perimeter and area.

### 2.9. Sholl Analysis

Images of fixed neurons transfected with sh-Scr-RFP were acquired with a confocal microscope (FV1000, Olympus, Tokyo, Japan), using a 40-oil immersion objective and excitation by a 555 nm laser (Biomedical Neuroscience Institute, Faculty of Medicine, Universidad de Chile). After the generation of z-projections, neurons were submitted to Sholl analysis using the ImageJ plugin software. The concentric radii started at 15 µm distance from the soma center, in 5 μm intervals. The following parameters were obtained: total intersections (i.e., the sum of all intersections with each different radius) and the maximum number of intersections (i.e., the maximum number of intersections reached by a neuron at any radius).

### 2.10. Lipid Peroxidation Measurement

After the respective treatments, lipid peroxidation was determined using the Bodipy C11 probe. To this aim, cultures were incubated for 30 min at 37 °C in the dark with 5 µM Bodipy C11. Then, cells were washed two times with PBS and were fixed for 15 min at 4 °C with 4% paraformaldehyde. After washing three times with PBS, the cells were labeled with the MAP2A antibody (to identify neurons), mounted on coverslips, and observed under the microscope. A confocal microscope (C2/C2si, NIKON) with a 40-oil immersion objective was used to acquire images by laser excitation at 405, 488, 561, and 640 nm. The ImageJ software was used for the generation of z-projections (sum of maximum intensity) from seven stacks (1 µm thickness each). Cells of interest were selected by delineation based on MAP2A staining; mean fluorescence intensity was measured for the green and red images, and the background was subtracted in both channels. Upon oxidation, the excitation maximum of the Bodipy C11 probe downshifts from 581 nm to 500 nm and the emission maximum from 591 nm to 510 nm. The oxidation ratio of C11 BODIPY 581/591 was calculated as an indicator of lipid peroxidation per cell following the equation:

Oxidation ratio = Bodipy^ox^/Bodipy^red^,

In this equation, Bodipy^red^ corresponds to the non-oxidized fraction of the probe and Bodipy^ox^ corresponds to the oxidized fraction.

### 2.11. Determination of Intracellular Ca^2+^ Signals

#### 2.11.1. Fluo4-AM

Primary hippocampal cultures, grown on 18 mm or 25 mm diameter glass plates, were transferred to a modified Tyrode solution (in mM: 129 NaCl, 5 KCl, 2 CaCl_2_, 1 MgCl_2_, 30 glucose, 25 HEPES-Tris, pH 7.3) and were incubated with 2 µM Fluo4-AM at 37 °C in the dark for 20 min. Subsequently, the cultures were washed two times with a modified Tyrode solution, and the cell-supporting glasses were mounted in a camera for microscopy analysis. In all cases, cells were placed at the microscope stage of a wide-field Zeiss Cell Observer epifluorescence microscope (Zeiss, REDECA, Faculty of Medicine, Universidad de Chile), using a 40x/1.00/W-DIC objective, 470-nm Colibri 2 light-emitting diode (LED)-based module, and a digital camera, electron-multiplying charge-coupled device (EMCCD) Evolve 512 delta (Teledyne Photometrics) with the Software ZEN Pro 2012. All settings were adjusted to minimize bleaching and maximize acquisition frequency. After recording a stable baseline, 10 mM caffeine or 5 μM thapsigargin were added to the culture. For analysis of Ca^2+^ signals, cells of interest were selected by hand using the ImageJ Software. The normalization of fluorescence signals, expressed as F/F_0_, was achieved using a semi-automatic protocol created in R-Script. It uses the table generated by ImageJ with the integrated intensity or the mean fluorescence measurement of the cells of interest as input. Moreover, if the records presented linear bleaching, this feature was corrected with another semi-automatic script designed for this purpose. The RStudio program (RStudio: Integrated Development for R. RStudio, PBC, Boston, MA) was used as an environment in the script running. These scripts are available at: https://github.com/ignacio-vegavasquez/supplementary-information (accessed on 9 February 2022).

#### 2.11.2. IncuCyte Live Imaging Measurements of Ca^2+^ Signals

Primary cultures were infected at 7–8 DIV with rAAV-GCaMP3-NES to induce the expression of GCaMP3 in the cytoplasm. After the respective treatments, cells were placed into an incubator with an incorporated IncuCyte S3 live-cell imaging apparatus (Sartorius) and four images per well were acquired in the green channel every hour for 24 h using a 20× objective. For the analysis, the IncuCyte Basic Analysis software was used. The background was subtracted, and objects were selected using the defined parameters of threshold and area.

### 2.12. Western Blot Analysis

In brief, cells were harvested and lysed in RIPA buffer containing protease inhibitor and phosphatase inhibitors. Next, samples were resolved by SDS-PAGE (4% polyacrylamide gels), transferred to PDVF membranes, and incubated overnight with specific antibodies against RyR2 (1:2000) or RyR3 (1:2000). Membranes were probed for GAPDH (1:20,000) as loading controls, and detection was performed using peroxidase-conjugated secondary antibodies. The ImageJ software was used to quantify optical band density.

### 2.13. Statistical Analysis

All data are expressed as mean ± SEM. The Shapiro–Wilk test was used to determine the normal distribution of the data, which were analyzed using One-Way-ANOVA followed by Tukey’s multiple comparison test. Two-way ANOVA followed by the Bonferroni post-hoc test was applied for multiple determinations.

## 3. Results

### 3.1. Inhibition of GPX4 by RSL3 Decreases Cell Viability in Primary Hippocampal Cultures

**Figure 1:**
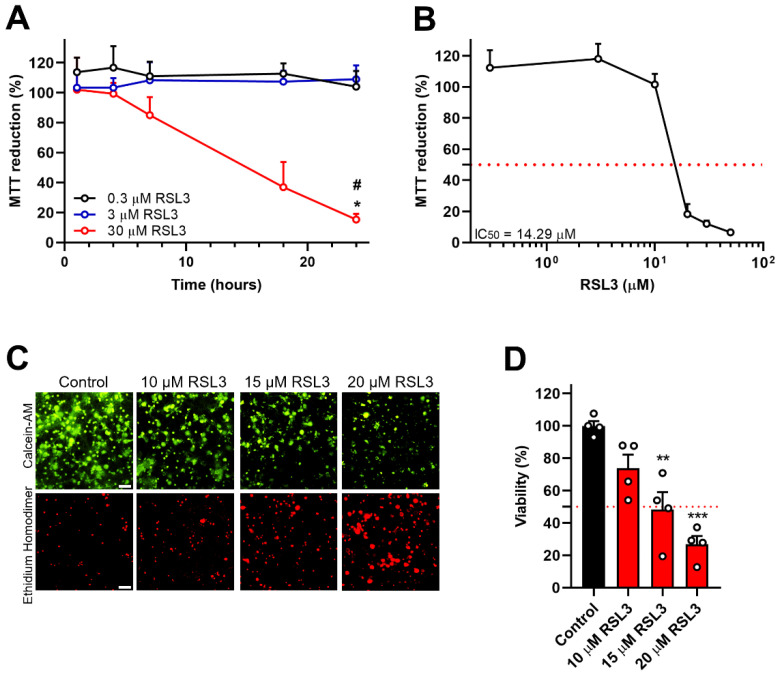
GPX4 inhibition by RSL3 decreases the cell viability of primary hippocampal cultures. (**A**). Time-concentration dependence. Primary hippocampal cultures were incubated with 0.3, 3, or 30 µM RSL3 for different times, and metabolic cell viability was assessed by the MTT assay. N = 3 independent experiments, with each condition tested in duplicates. Statistically significant differences among experimental conditions were evaluated by Two-Way ANOVA followed by a Bonferroni post-hoc test. * *p* < 0.0332 compared to the 0.3 µM RSL3 condition, # *p* < 0.0332 compared to the 3 µM RSL3 condition. (**B**). IC_50_ definition. Primary hippocampal neurons were incubated for 24 h with different concentrations of RSL3, and metabolic cell viability was evaluated by the MTT assay. The IC_50_ at 24-h incubation with RSL3 was 14.29 µM. (**C**). LIVE/DEAD Viability/Toxicity Kit. Left: Representative images of primary hippocampal cultures incubated with DMSO (control) or 10, 15, or 20 µM RSL3 for 24 h and stained with calcein-AM (green) and ethidium homodimer (red). Scale Bar 50 µm. (**D**). Quantification of calcein positive cells, per the sum of calcein and ethidium homodimer positive cells, which reveals an IC_50_ of 15 µM. In (**B**,**D**), values were normalized to control. Minimum N = 3 independent experiments, with each condition performed in triplicates. Statistically significant differences among experimental conditions were evaluated by One-Way ANOVA followed by Tukey’s multiple comparisons test. ** *p* < 0.0021, *** *p* < 0.0002 compared to control.

To evaluate the potential role of ferroptosis on neuronal viability, cultured hippocampal neurons were incubated for different times with increasing concentrations of the GPX4 inhibitor RSL3; these treatments revealed that RSL3 provoked time and concentration-dependent decreases in cell viability (Figure 1A). Next, the effects of GPX4 inhibition by varying RSL3 concentrations were evaluated in 24-h incubation periods (Figure 1B). Different viability assays (MTT assay, LIVE/DEAD Viability/Cytotoxicity Kit) showed that incubation for 24 h with 14.3 µM RSL3 induced 50% cell death (IC_50_) (Figure 1B–D).

### 3.2. GPX4 Inhibition by RSL3 Induces Ferroptosis in Primary Hippocampal Cultures

**Figure 2:**
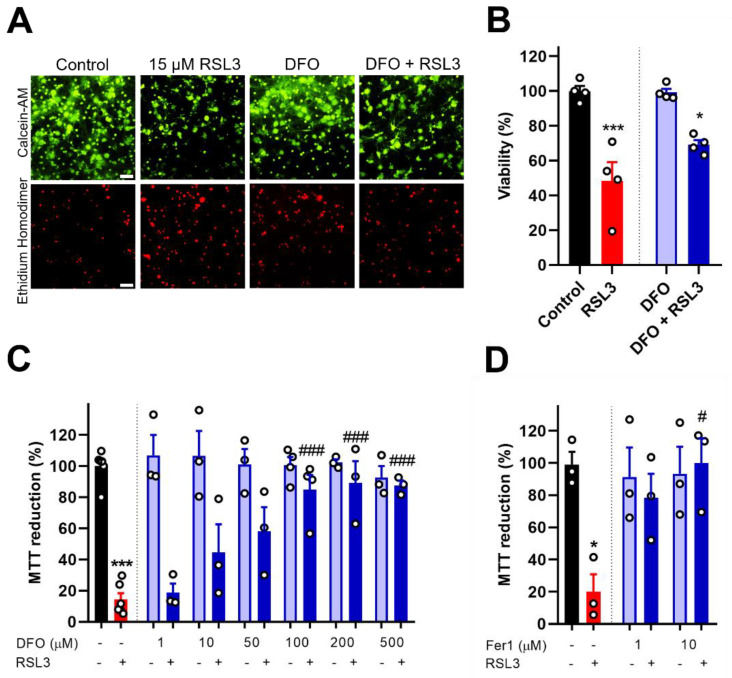

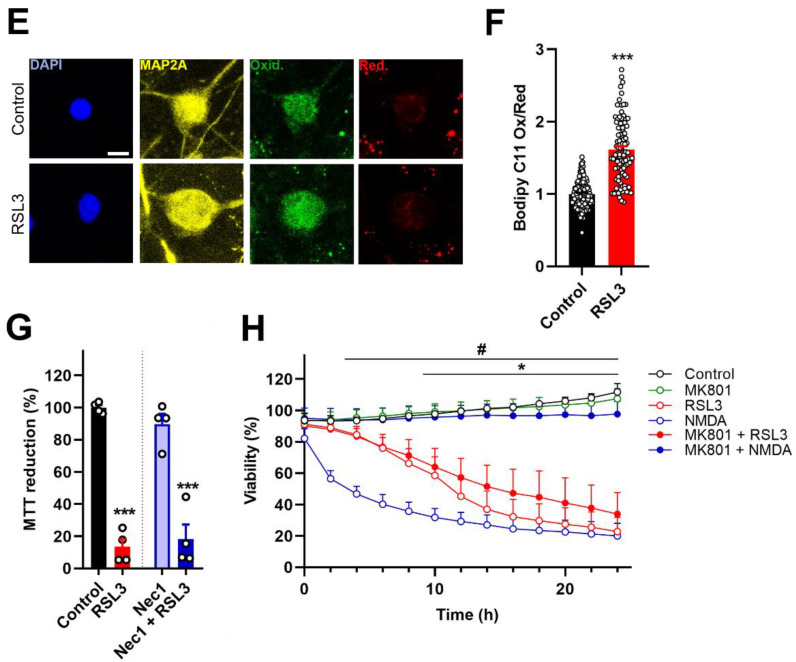
GPX4 inhibition by RSL3 induces ferroptosis in primary hippocampal cultures. (**A**–**C**). Effects of DFO. (**A**,**B**). Primary hippocampal cultures were preincubated for 6 h with 100 µM DFO, followed by a 24-h incubation of 15 µM RSL3. Cell viability was assessed by LIVE/DEAD Viability Cytotoxicity Assay. (**A**). Representative images of primary hippocampal cultures are shown by calcein-AM and ethidium homodimer staining. Scale Bar 50 µm. (**B**). Quantification of calcein-positive cells over the sum of calcein- and ethidium-homodimer-positive cells. N = 4 independent experiments, with each condition performed in triplicates. (**C**). Primary hippocampal cultures were preincubated for 6 h with 1, 10, 50, 100, 200, or 500 µM DFO, followed by a 24-h incubation of 15 µM RSL3. Cell viability was assessed by MTT assay. Minimum N = 3 independent experiments, with each condition performed in triplicate. (**D**). Effects of Fer1. Primary hippocampal cultures were preincubated for 6 h with 1 or 10 µM Fer1 followed by 24-h incubation with 15 µM RSL3. Cell viability was determined by the MTT assay. N = 3 independent primary hippocampal cultures; each condition was done in duplicates or triplicates. (**E**). Representative confocal microscopy images of a primary hippocampal neuron were treated with DMSO (control) or RSL3. Cells were labeled with DAPI to stain the nucleus (blue), with MAP2A antibody (yellow), and Bodipy C11 (red: reduced, green: oxidized). Z-Projection of Maximum Intensity. Scale bar 10 µM. (**F**). Quantification of the ratio of green/red fluorescence intensity showing individual cells per condition (N = 5 independent cell cultures). Statistically significant differences among experimental conditions were evaluated by unpaired t-test. (**G**). Effects of Nec1. Primary hippocampal cultures were preincubated for 2 h with 50 µM Nec1 dissolved in DMSO (final concentration, 0.25%) followed by a 24-h incubation period with 15 µM RSL3. Cell viability was determined by the MTT assay. N = 4 independent primary hippocampal cultures with each condition in duplicates or triplicates. (**H**). Effects of MK801. Primary hippocampal rat cultures were preincubated for 1 h with 10 µM MK801, followed by 24-h incubation with 15 µM RSL3 or 30 µM NMDA. Cell viability was assessed by monitoring the time-dependent changes in the red fluorescence of cells infected with rAAV-mCherry-NLS. Images were acquired with the IncuCyte S3 live-cell imaging apparatus (Sartorius) using a 20x objective. Minimum N = 3 independent hippocampal cultures. Primary hippocampal cultures from P1 pups. Statistically significant differences among experimental conditions were evaluated by Two-Way ANOVA followed by a Bonferroni post-hoc test. * *p* < 0.0332 RSL3 compared to DMSO (control). # *p* < 0.0332 NMDA compared to DMSO (control). For MTT and LIVE/DEAD Assay, statistically significant differences among experimental conditions were evaluated by One-Way ANOVA followed by Tukey’s multiple comparisons test. * *p* < 0.0332, *** *p* < 0.0002 relative to control. # *p* < 0.0332, ### *p* < 0.0002 relative to neurons treated with RSL3.

To further test if RSL3 treatment induced ferroptosis, primary hippocampal cultures were co-incubated with the iron chelator DFO or the ferroptosis inhibitor Fer1. Both the LIVE/DEAD Viability/Cytotoxicity Kit (Figure 2A,B) and the MTT assay (Figure 2C,D) revealed that the decrease in cell viability induced by RSL3 treatment was mitigated by previous incubation with DFO or Fer1. These protective effects positively correlated with increased DFO or Fer1 concentrations. Moreover, since lipid peroxidation is one of the hallmarks of ferroptosis, we tested with the redox-sensitive probe Bodipy C11 if RSL3 treatment increased lipid peroxidation. In the basal state, this probe displays red fluorescence (Em 590 nm); however, when its polyunsaturated butadienyl zone gets oxidized, its emission/excitation pattern changes towards green fluorescence (Em 510 nm). We found that after 24 h of RSL3-treatment, MAP2A-stained neurons displayed a significant increase in the ratio of green to red fluorescence of Bodipy C11 compared to the controls (Figure 2E,F), an indication of increased lipid peroxidation.

In addition, we evaluated if RSL3 treatment triggered the necrotic or excitotoxic pathways. Our results showed that RSL3-induced cell death was not mitigated by the necrosis inhibitor Necrostatin-1 (Nec1), which was dissolved in DMSO (Figure 2G); hence, neither 50 µM Nec1 nor 0.25% DMSO influenced RSL3-induced cell death. Moreover, the NMDA receptor inhibitor MK801 effectively protected against NMDA toxicity, but not against RSL3-mediated cell death (Figure 2H). Based on these results, we suggest that RSL3-induced cell death does not engage components of the necrotic pathway or the activity of NMDA receptors. Overall, these results confirm that RSL3 treatment induced ferroptosis, but not necrosis or excitotoxicity in primary hippocampal cultures.

### 3.3. Ferroptosis Induces Cell Swelling and Reduces Dendritic Complexity in Primary Hippocampal Neurons

**Figure 3:**
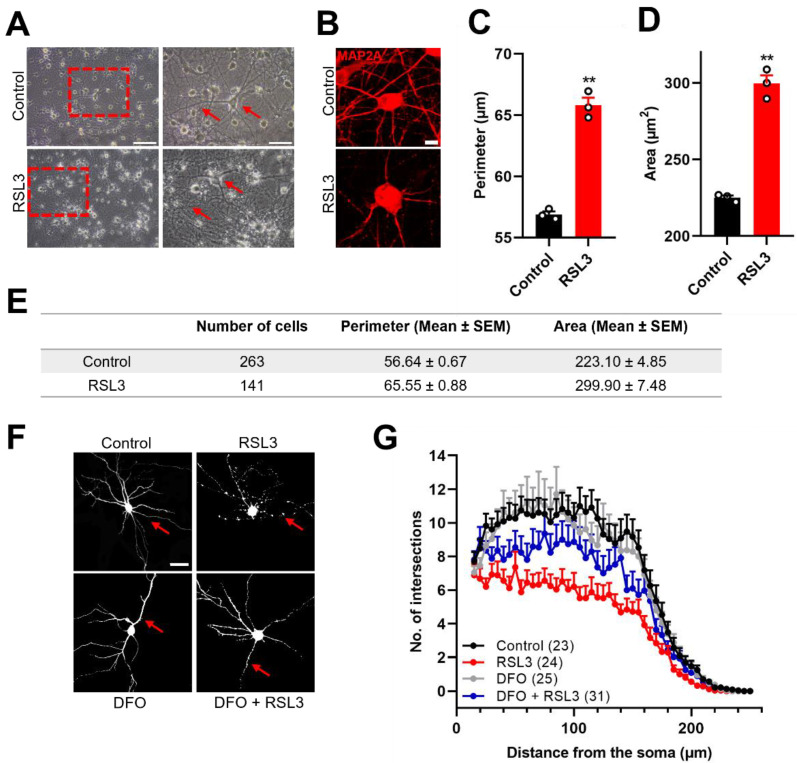

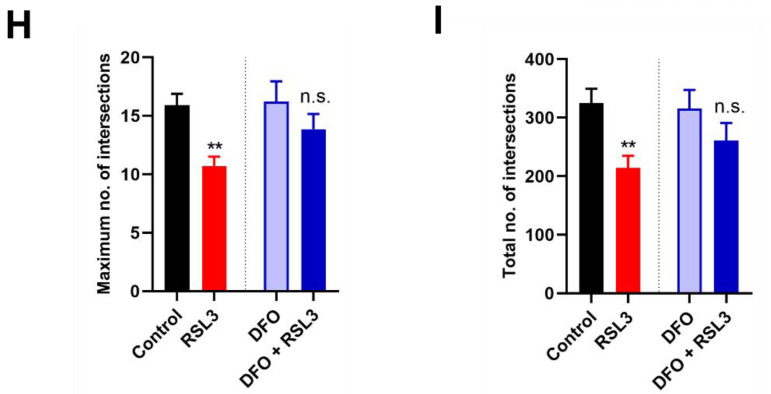
Ferroptosis induces cell swelling and reduces dendritic complexity in primary hippocampal cultures. (**A**). Representative light microscopy images showing a control neuron treated with DMSO (control) and a neuron treated with RSL3, displaying cell swelling and dendritic damage (indicated by red arrows). Scale bar 100 µm (left) and 50 µm (right). (**B**). Representative confocal microscopy images of primary hippocampal cultures treated with DMSO (control) or RSL3 and labeled with MAP2A antibody. Scale bar 10 µM. (**C**,**D**). Quantification of soma perimeter and area showing the mean of each independent hippocampal culture. Statistically significant differences among experimental conditions were evaluated using a paired t-test. (**E**). Quantification of soma perimeter and area of individual cells; N = 3 independent hippocampal cultures. (**F**). Representative images of hippocampal neurons transfected with RFP for morphological analyses. Scale Bar: 50 µm. (**G**). *Sholl* Analysis of neurons treated with DMSO (control), DFO, RSL3, or DFO + RSL3. (**H**,**I**). Quantification of morphological parameters. (**H**). Maximum number of intersections and (**I**). total number of intersections for DMSO (control); n = 23; DFO, n = 24; RSL3, n = 25; DFO + RSL3, n = 31. N = 3 independent experiments. Statistically significant differences among experimental conditions were evaluated by One-Way ANOVA followed by Tukey’s multiple comparison test. ** *p* < 0.0021, n.s. not significant.

Next, the cell morphology of primary hippocampal cultures treated with RSL3 was evaluated. The light microscopy images of primary hippocampal neurons revealed morphological changes (swelling of the soma and dendritic damage) highlighted by red arrows in RSL3-treated neurons compared to the control (Figure 3A,B). The quantification of the soma perimeter and area of MAP2A-stained hippocampal neurons after ferroptosis induction revealed a significant increase of 16% in the perimeter of the soma, and of 34% in the area of the soma (Figure 3C–E).

We next analyzed the effects of RSL3 on neuronal dendritic complexity. To this aim, neurons were transfected with an RFP probe and treated with the ferroptosis inducer RSL3, plus or minus DFO; *Sholl* Analysis was used to quantify the dendritic complexity. Figure 3F,G show that RSL3 drastically decreased dendritic complexity compared to control neurons. The quantification of morphological parameters, such as the maximum number of intersections and the total number of intersections, revealed the significant inhibitory effects of the ferroptosis inducer RSL3 on dendritic complexity (Figure 3H,I). Interestingly, these injurious changes were diminished in neurons preincubated with DFO and subsequently treated with RSL3, confirming that iron chelation protects against the dendritic damage induced by ferroptosis (Figure 3F, indicated by red arrows).

### 3.4. Ferroptosis Provokes a Time-Dependent Increase in Cytoplasmic Ca^2+^

**Figure 4:**
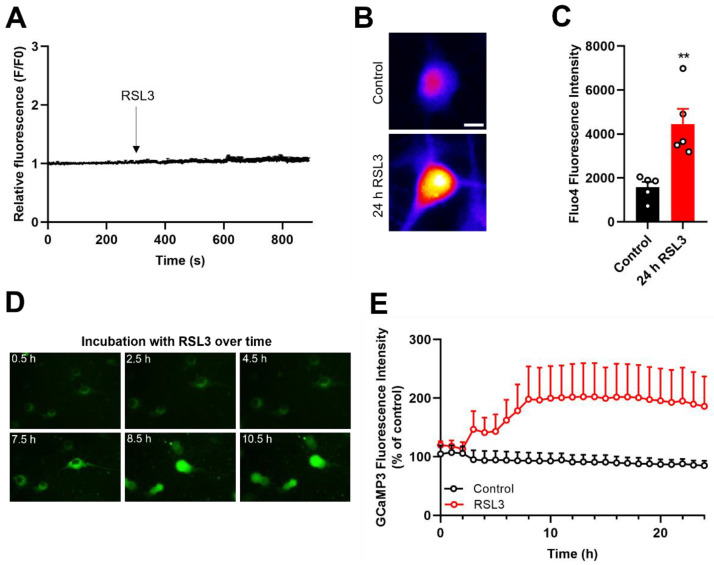
RSL3 treatment induces cytoplasmic Ca^2+^ increases over time. (**A**). Representative time course of Fluo4 fluorescence changes before and after the addition of 15 μM RSL3 to primary hippocampal cultures. Similar findings were obtained in N = 3 independent hippocampal cultures. (**B**,**C**). Primary hippocampal neurons treated with DMSO (control) or 15 μM RSL3 (24 h) were loaded with Fluo4, and basal fluorescence was recorded. (**B**). Representative images of DMSO (control) and RSL3 treated neurons. Scale bar 10 µm. (**C**). Quantification of the basal Fluo4 fluorescence displayed by N = 5 independent hippocampal cultures after 24 h incubation with 15 μM RSL3 or DMSO (control). Statistically significant differences among experimental conditions were evaluated by paired t test. ** *p* < 0.0021. (**D**). Representative images of primary hippocampal cultures infected with rAAV-GCaMP3-NES. Images were acquired with the IncuCyte S3 live-cell imaging apparatus (Sartorius) using a 20× objective. (**E**). Primary hippocampal cultures infected with rAAV-GCaMP3-NES showed time-dependent cytoplasmic [Ca^2+^] increases in RSL3 treated hippocampal cultures. Primary hippocampal cultures from P1 pups were used in these experiments.

We investigated next the possible participation of Ca^2+^ signaling to RSL3-induced ferroptosis. To determine cytoplasmic changes in Ca^2+^ concentration, cells were loaded with the green fluorescent probe Fluo4-AM (Kd = 335 nM, Ex/Em maxima: 494/506 nm). As illustrated in Figure 4A, RSL3 addition to primary hippocampal cultures did not induce an acute increase in cytoplasmic [Ca^2+^]. However, hippocampal cultures preincubated with RSL3 for 24 h, and then loaded with Fluo4-AM displayed a significant increase in fluorescence intensity compared to the controls (Figure 4B,C). Next, we investigated the temporality of these [Ca^2+^] increments. To this aim, cultures were transduced with the cytoplasmic Ca^2+^ probe GCaMP3-NES, which offers some advantages over the chemical Ca^2+^ probe Fluo4, such as precise targeting into neurons and more prolonged image detection. As evidenced by the GCaMP3-NES fluorescence, two hours after RSL3 addition, neurons displayed a significant increase in cytoplasmic [Ca^2+^], which reached a plateau after a few hours and was maintained onwards (Figure 4D,E).

### 3.5. RyR-Mediated Ca^2+^ Release Contributes to Ferroptosis

**Figure 5:**
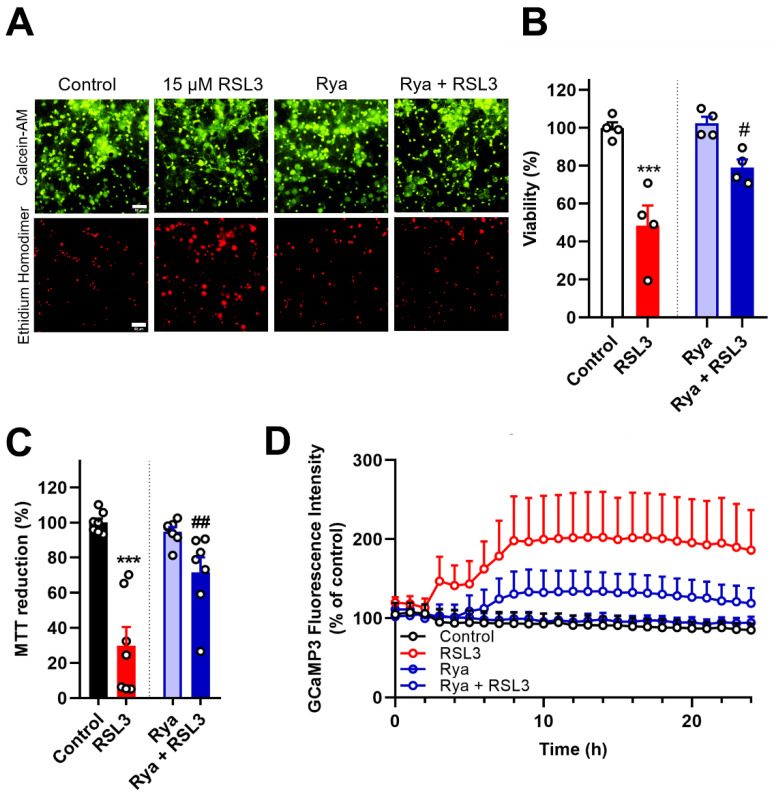
RyR channel inhibition partially protects against ferroptosis and reduces the increase in cytoplasmic [Ca^2+^] induced by RSL3 treatment. (**A**,**B**). Primary hippocampal cultures were preincubated for 1 hour with 20 µM Rya to suppress RyR activity followed by 24-hour incubation with 15 µM RSL3. Cell viability was assessed by the LIVE/DEAD Viability Cytotoxicity Assay. (**A**). Representative images of primary hippocampal cultures loaded with calcein-AM (green) and stained with ethidium homodimer (red). Scale Bar 50 µm. (**B**). Quantification of calcein-positive cells, over the sum of calcein- plus ethidium homodimer-positive cells, reveals the partial protective effects of RyR inhibition on RSL3-induced ferroptosis. N = 4 independent experiments, with each condition performed in triplicate. (**C**). Primary hippocampal cultures were preincubated for 1 hour with 20 µM ryanodine (Rya) followed by 24-hour incubation with 15 µM RSL3. Metabolic cell viability was assessed by the MTT assay. N = 7 independent experiments; each condition was done in duplicate or triplicate. (**D**). Primary hippocampal cultures infected with rAAV-GCaMP3-NES showed that RSL3 induced time-dependent cytoplasmic [Ca^2+^] increases, which were reduced by preincubation with 20 µM Rya. Images were acquired with the IncuCyte S3 live-cell imaging apparatus (Sartorius) using a 20× objective. Primary hippocampal cultures were from P1 pups. Statistically significant differences among experimental conditions were evaluated by One-Way ANOVA followed by Tukey’s multiple comparisons test. *** *p* < 0.0002 relative to DMSO (control). # *p* < 0.0332, ## *p* < 0.0021 relative to neurons treated with RSL3.

Based on the results described above, next we tested if the increase in cytoplasmic [Ca^2+^] during RSL3 treatment arises through Ca^2+^ release mediated by the ER-resident RyR channels, which are expressed at higher levels than IP_3_R channels in the rat hippocampus [30], and contribute to ferroptosis in microglial cells [28]. To test the effects of RyR-mediated Ca^2+^ release on ferroptosis, primary hippocampal cultures were incubated for 1 h with a concentration of ryanodine (20 µM) that eliminates RyR activity prior to RSL3 treatment [34]. Cell viability was then evaluated via the LIVE/DEAD Viability/Cytotoxicity Assay (Figure 5A,B) and the MTT assay (Figure 5C). The results revealed that suppression of RyR activity, which did not cause cell death, partially protected against ferroptosis induced by 24-h incubation with 15 µM RSL3. Moreover, as illustrated in Figure 5D, the increase in cytoplasmic [Ca^2+^] induced by RSL3 treatment was reduced in conditions of RyR activity suppression, suggesting that the [Ca^2+^] increase induced by RSL3 is partly mediated by RyR channel activation. Thus, we can conclude that Ca^2+^ release from the ER-mediated by RyR channels participates in RSL3-induced ferroptosis.

### 3.6. RSL3 Treatment Reduces the Response to Caffeine and Thapsigargin and Decreases RyR2 Protein Content

**Figure 6:**
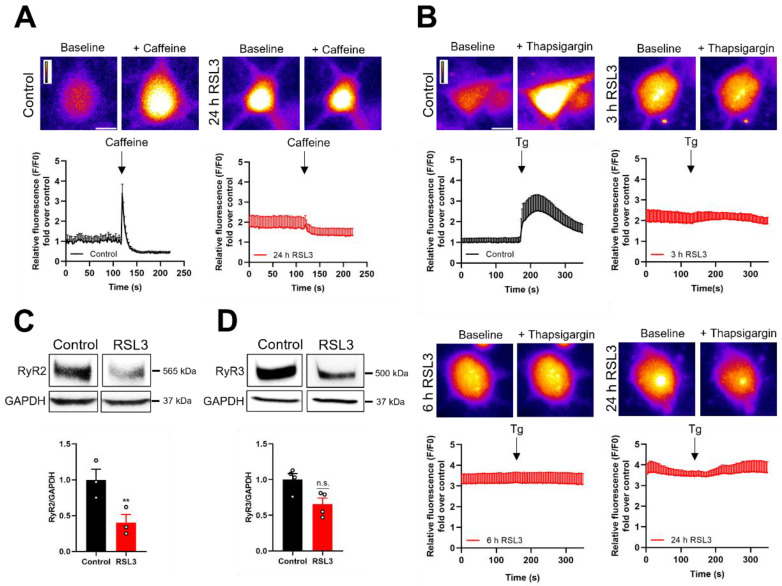
RSL3 decreases the response to caffeine and thapsigargin (Tg) and reduces RyR2 protein content. (**A**,**B**). Primary hippocampal cultures treated with DMSO (control) or RSL3 were loaded with Fluo4, and fluorescence was recorded in Ca^2+^-free Tyrode solution before and after the addition of 10 mM caffeine. (**A**). Upper panels: Representative images of DMSO (control) and RSL3 (24 h) treated hippocampal cultures before and after the addition of 10 mM caffeine. Scale bar 10 µm. Lower panels: Plot of the fluorescence intensity ratio over basal fluorescence recorded in each condition, before and after the addition of 10 mM caffeine. Similar findings were obtained in N = 3 independent hippocampal cultures. (**B**). Primary hippocampal cultures treated with DMSO (control) or RSL3 (3 h, 6 h, 24 h) were loaded with Fluo4, and fluorescence was recorded in a Ca^2+^ free Tyrode solution before and after the addition of 5 µM thapsigargin. Upper panels: Representative images of DMSO (control) or RSL3 treated hippocampal cultures before and after adding thapsigargin. Scale bar 10 µm. Lower panels**:** Plot of the fluorescence intensity ratio over the basal fluorescence displayed in each case, before and after the addition of 5 µM thapsigargin (arrow). Similar findings were obtained in N = 3 independent hippocampal cultures. (**C**,**D**). Representative Western blots, showing RyR2 and RyR3 protein contents and quantification of RyR2 and RyR3 protein contents, respectively, normalized to GAPDH. Minimum N = 3 independent hippocampal cultures. Statistically significant differences among experimental conditions were evaluated by paired t-test. ** *p* < 0.0021, n.s. not significant.

Considering the previous results, we tested if caffeine, a well-known RyR agonist, evokes an increase in Fluo4 fluorescence intensity via Ca^2+^ efflux from the ER in RSL3-treated cultures. We found that cultures treated with RSL3 for 24 h, which displayed higher basal fluorescence levels than controls, did not respond to the addition of 10 mM caffeine (Figure 6A). The lack of response to caffeine displayed by neurons treated with RSL3 for 24 h suggests that this treatment depleted the ER of releasable Ca^2+^. Accordingly, next we tested if SERCA inhibition with thapsigargin evoked an increase in Fluo4 fluorescence intensity as a result of net Ca^2+^ efflux from the ER. We found that cultures treated with RSL3 for 3 h, 6 h, or 24 h did not present the fluorescence increase displayed by control cultures following the addition of 5 µM thapsigargin (Figure 6B). Hence, we propose that treatment with RSL3 depletes the ER of releasable Ca^2+^. In addition, RSL3 treatment for 24 h significantly decreased RyR2 protein contents in primary hippocampal cultures (Figure 6C,D).

## 4. Discussion

### 4.1. GPX4 Inhibition by RSL3 Leads to Ferroptosis in Primary Hippocampal Cultures

Previous reports have described ferroptosis in neuronal cell lines and in vivo models and have elucidated several signaling pathways that govern ferroptosis [35]. However, the characterization of ferroptosis in primary hippocampal cultures, as well as the role of Ca^2+^, principally its contribution via release from the ER through RyR channels, has not been reported to date. Hence, we addressed these subjects using in vitro primary cultures of rat hippocampal neurons. To induce ferroptosis, we used RSL3, an agent that inhibits GPX4—an enzyme with a crucial role in the control of ferroptosis [36].

The novel results presented in this work established an in vitro RSL3-induced ferroptosis model using primary hippocampal neurons from rat embryos. Thus, RSL3-mediated GPX4 inhibition decreased the viability of primary hippocampal cultures in a dose- and time-dependent manner. A concentration of 15 µM RSL3 resulted in 50% cell death (IC_50_) after 24 h of incubation, in agreement with the previously described IC_50_ (12.15 µM) for RSL3 in primary cortical neurons [37]. Moreover, our studies evidenced that the ferroptosis inhibitors DFO and Fer1 effectively protected against RSL3-induced cell death in primary hippocampal cultures. In contrast, the necrosis inhibitor Nec1 failed to do so, confirming that RSL3-treatment did not elicit necrosis.

Pathologically high extracellular levels of the neurotransmitter glutamate triggers excitotoxicity through the excessive activation of glutamate receptors, leading to neuronal death [37,38,39,40,41]. Excitotoxicity has been increasingly implicated in traumatic brain injury and neurodegenerative diseases. Excitotoxicity and ferroptosis seem to display shared mechanisms. Both excitotoxicity and ferroptosis can be induced by glutamate, whereas ferroptosis inhibitors protect against glutamate-induced excitotoxicity [1]. In addition, excitotoxicity also seems to involve lipid peroxidation, as evidenced in NMDA-treated primary hippocampal neurons [42]. Hence, we tested if NMDA receptor inhibition protected against RSL3-induced ferroptosis. Our results show that the NMDA receptor antagonist MK801 did not protect against the lethal effects of RSL3, confirming that RSL3 treatment does not involve NMDA-receptor-related pathways, as does excitotoxicity.

In addition, we report that ferroptosis induction involved morphological changes in primary hippocampal cultures, evidenced by the increases in the perimeter and the area of the soma induced by RSL3. These results are in concordance with studies in HeLa cells that describe “cell swelling” as one of the morphological hallmarks of ferroptosis [43,44]. Moreover, primary hippocampal neurons presented dendritic damage suggestive of a dying-back mechanism [45], which was reduced by previous DFO incubation. These findings might provide insights into the steps leading to ferroptosis, which possibly begin at the dendrites and then proceed to the soma. These deleterious effects could significantly impair crucial neuronal functions such as synapsis formation and synaptic plasticity.

Overall, these results confirm ferroptosis as a possible cell death mechanism that mediates neuronal death in the hippocampus, and which may play an important role in neurodegenerative diseases such as AD.

### 4.2. RyR-Mediated Ca^2+^ Release Contributes to RSL3-Induced Ferroptosis in Primary Hippocampal Neurons

Regulated Ca^2+^ signals are relevant to maintain metabolic and essential neuronal functions, as well as the survival of neurons. Disruption of neuronal Ca^2+^ homeostasis promotes the ensuing deregulation of Ca^2+^-dependent signaling pathways, which can be lethal. Considering that the ER is the most prominent intracellular Ca^2+^ reservoir, and that RyR channels are highly redox sensitive [20,27], the increase in ROS associated with ferroptosis may lead to substantial RyR activation, which could cause abnormally high and noxious increases in cytoplasmic and mitochondrial [Ca^2+^]. Despite a growing number of reports targeting ferroptosis and elucidating its mechanisms, the role of Ca^2+^ in ferroptosis is still controversial.

Using cytoplasmic fluorescent Ca^2+^ indicators, we could observe that cytoplasmic [Ca^2+^] started to increase two hours after RSL3 addition. Concordantly, a recent study in mouse fibroblasts detected a significant increase in cytoplasmic [Ca^2+^] after one hour of treatment with RSL3, which was inhibited by the ferroptosis inhibitor Fer1 [46]. To evaluate if the increase in cytoplasmic [Ca^2+^] originated through Ca^2+^ release from the ER, cultures were incubated with a concentration of ryanodine that suppresses RyR activity without decreasing ER calcium content [34]. This condition partially protected against RSL3-induced ferroptosis in primary hippocampal neurons. Although the slow-surging increase in cytoplasmic [Ca^2+^] provoked by ferroptosis was reduced by prior inhibition of RyR channel activity, it was not completely suppressed, indicating that sources other than RyR-mediated Ca^2+^ release give rise to these cytoplasmic [Ca^2+^] increments. Previous studies have shown that SOCE-mediated Ca^2+^ influx, which takes place after the depletion of Ca^2+^ within the ER, enhances ferroptosis [47,48,49]. Future studies should address if the SOCE pathway contributes to the delayed increase in cytoplasmic [Ca^2+^] induced by RSL3 in primary hippocampal neurons.

Additionally, we report here that drugs such as caffeine and thapsigargin failed to provoke ER Ca^2+^ release from neurons undergoing ferroptosis in response to treatment with RSL3 for 24 h; indeed, thapsigargin addition did not cause net ER Ca^2+^ release after incubation of RSL3 for 3 h or 6 h, confirming that Ca^2+^ release from the ER contributes to the significant increase in cytoplasmic [Ca^2+^] in the initiating steps of ferroptosis. These results provide evidence that neuronal ER Ca^2+^ stores are depleted through excessive RyR-mediated Ca^2+^ release—and possibly via SERCA inhibition—caused by the increased oxidative tone induced by GPX4 inhibition. Of note, primary hippocampal cultures displayed lower levels of RyR2 protein contents, which might be a protective mechanism against the excessive release of Ca^2+^ mediated by RyR channels. Interestingly, the decrease in RyR protein content was higher for the RyR2 than for the RyR3 channel isoform, which is the principal RyR isoform expressed in hippocampal neurons from rodent tissues [30]. In line with this notion, a recent study in microglia reported that RyR-mediated Ca^2+^ release contributes to ferroptosis induced by nitrogen-doped graphene quantum dots triggering the ER stress response [28]. However, assessing if RSL3 treatment activates the ER stress response in primary hippocampal neurons needs further investigation. The role of ER-mediated Ca^2+^ signals in ferroptosis has also been recently described in different cell lines, indicating that luminal Ca^2+^ stores alter the sensitivity to ferroptosis by inducing lipid remodeling, specifically by altering lipid elongation and saturation state [26]. This finding confirms our hypothesis that ER-mediated Ca^2+^ signals play a significant role in the initial and the executive steps of ferroptosis.

This study provides the first integrative evidence that Ca^2+^ release from the ER via RyR channels mediates ferroptosis induced by GPX4 inhibition in primary hippocampal neurons. There is growing evidence that the relationship between Ca^2+^ and iron is Janus-faced because excessive iron loads can alter cellular [Ca^2+^] above homeostatic levels, therefore promoting the progression of neuronal death in neurodegenerative diseases [50,51].

To conclude, the present work reveals Ca^2+^-mediated signals as a promising target for novel therapeutic intervention strategies and possibly provides the necessary framework for developing inhibitors of ferroptosis to treat neurological diseases.

## 5. Conclusions

Our studies have shown (1) that ferroptosis might be one of the cell death types involved in hippocampal-related diseases, and (2) that RyR-mediated Ca^2+^ signals contribute to ferroptosis in primary hippocampal neurons (see Graphical Abstract). Increasing cellular [Ca^2+^] above homeostatic levels can have deleterious effects on proper neuronal function. Therefore, future studies are highly relevant to understand the dynamics of Ca^2+^ signaling in hippocampal neuronal death.

## Data Availability

All data are presented in the figures. Raw data are available upon request.
